# Cracked Tooth: A Report of Two Cases and Role of Cone Beam Computed Tomography in Diagnosis

**DOI:** 10.1155/2012/525364

**Published:** 2012-11-06

**Authors:** Pishipati Vinayak Kalyan Chakravarthy, Lahari Ajay Telang, Jayashri Nerali, Ajay Telang

**Affiliations:** ^1^Department of Conservative Dentistry and Endodontics, Penang International Dental College, Level 18-21, NB Tower, 5050 Jalan Bagan Luar, 12000 Butterworth, Penang, Malaysia; ^2^Department of Oral Medicine and Radiology, Penang International Dental College, 12000 Butterworth, Penang, Malaysia; ^3^Department of Oral Pathology, Penang International Dental College, 12000 Butterworth, Penang, Malaysia

## Abstract

Cracked tooth is a distinct type of longitudinal tooth fracture which occurs very commonly and its diagnosis can be challenging. This type of fracture tends to grow and change over time. Clinical diagnosis is difficult because the signs and symptoms are variable or nonspecific and may even resemble post-treatment disease following root canal treatment or periodontal disease. This variety and unpredictability make the cracked tooth a challenging diagnostic entity. The use of cone beam computed tomography (CBCT) in diagnosis of complex endodontic cases has been well documented in the literature. In this paper we present two cases of cracked tooth and emphasise on the timely use of cone beam computed tomography as an aid in diagnosis and as a prognostic determinant.

## 1. Introduction

Longitudinal tooth fractures are characterized by an incomplete or complete fracture line that extends through the long axis of the tooth [1]. These linear fractures tend to grow and change over time [2]. They have been classified into 5 distinct groups, generally from least to most severe: craze lines; fractured cusp; cracked tooth; split tooth; vertical root fractures [3].

Cracked tooth is a distinct type of longitudinal fracture which may extend through either or both of the marginal ridges and through the proximal surface. The fracture may be restricted to the crown or may extend from crown to the proximal root for varying distances [3].

Cracked tooth is common and challenging [4]. It may be caused by excessive forces from mastication or occlusion, either large forces on a normal tooth or normal forces on a weakened tooth [1]. Complex restorative and endodontic treatments that remove dentin compromise the internal strength of the tooth making it susceptible to fracture [3]. The detection of non-displaced longitudinal fractures, such as a cracked tooth, is a significant challenge in clinical practice [4]. Clinical diagnosis is difficult because the signs and symptoms are variable or nonspecific and may even resemble post-treatment disease following root canal treatment or periodontal disease [5]. Radiographic signs are usually absent when the orientation of the X-ray beam is not parallel to the plane of the fracture making the diagnosis even more challenging [6]. Moreover superimposition of other structures further limits the sensitivity of radiographs for the detection of fractures.

Cracked tooth by itself is not a diagnosis, but is a finding. A cracked tooth can act as a pathway for bacteria that may induce pulpal and/or periapical inflammation or disease. The relationship between cracks in teeth and endodontic diagnosis depends upon the extent of the fracture. If the fracture is in or in close proximity to the pulp and allows bacterial byproducts or frank bacteria to communicate with the pulp, then inflammation and pulpal degeneration occurs. If the fracture is not in close proximity to the pulp and bacterial byproducts are neutralized in the dentinal tubules, then no pulpal inflammation or degeneration should be expected [7]. The prognosis of a tooth depends on extent of the fracture. The prognosis of cracked tooth that is not treated will progressively deteriorate and may evolve into a split tooth or result in severe periodontal defects [1]. Eventually the tooth may be lost. Therefore early diagnosis and treatment are essential in saving these teeth.

In this paper two case reports of cracked tooth which reported to the dental clinic of Penang International Dental College have been presented. The value of cone beam CT as a prognostic determinant has been highlighted.

## 2. Case Report 1

**Figure 1:**
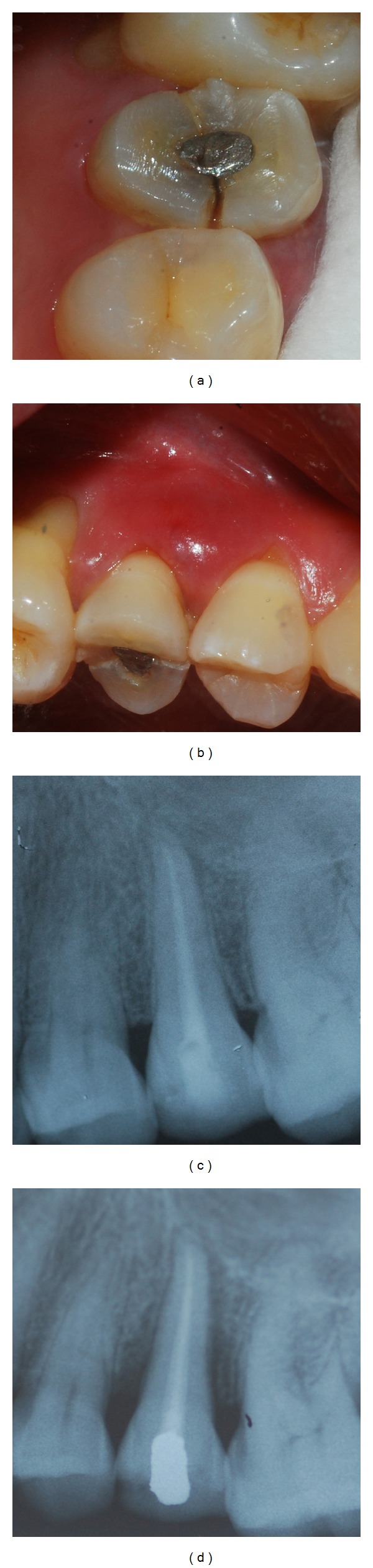
(a) Fracture extending mesiodistally on centre of occlusal surface and then onto proximal surface of 25. (b) Diffuse swelling of the buccal gingiva. (c) Post obturation intraoral periapical radiograph of 25. (d) Intraoral periapical radiograph taken after 15 months shows severe angular bone loss interproximally associated with propagation of fracture line.

A female patient aged 49 years reported with pain on upper left second premolar, that is, 25, for the past two weeks. Pain was severe, throbbing in nature, and radiating towards the temporal region and the neck. It was aggravated on taking cold and hot drinks and relieved with pain killers (NSAIDs). No caries or fracture was detected nor was restoration present. The tooth was tender on percussion and no mobility was detected. Electric pulp test showed delayed response. Radiograph revealed no significant findings. Based on the history and clinical presentation it was diagnosed as apical periodontitis. Root canal treatment was performed with amalgam as post-endodontic restoration (Figure 1(c)). Thereafter, the patient failed to report for a crown restoration.

Fifteen months later, the patient returned with severe pain in the same tooth, that is, 25. Pain was radiating and aggravated on chewing and biting. Patient could recall episodes of dull aching pain even after the root canal therapy, for which she was taking pain killers for relief. Clinical examination revealed a fracture on the centre of occlusal surface running mesiodistally and over the marginal ridges onto the proximal surface (Figure 1(a)). A diffuse swelling was present on the buccal gingiva involving the marginal and attached gingiva (Figure 1(b)). Tooth was tender on percussion and exhibited grade II mobility. The fracture could not be separated by using wedging forces. Periodontal probing revealed deep isolated narrow pockets on both proximal sides. Radiograph did not reveal the fracture as it was mesiodistally oriented, but showed angular bone loss on both mesial and distal proximal aspects and generalised widening of periodontal ligament space (Figure 1(d)). Prognosis was considered poor and extraction was performed.

**Figure 2:**
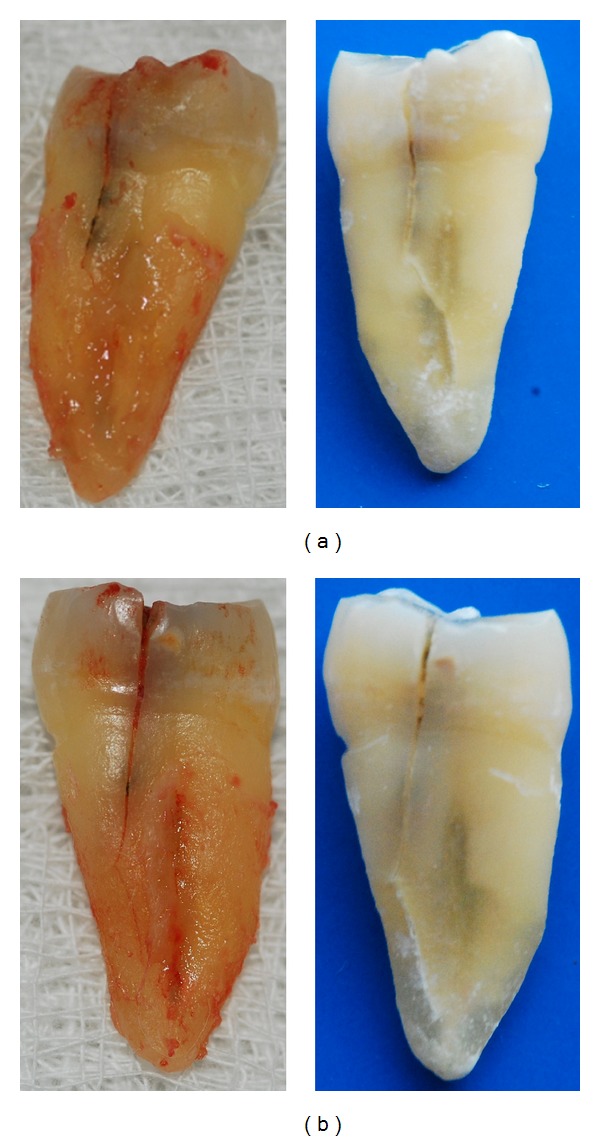
(a) Fracture extending to junction of middle and apical third of root (mesial) of 25. (b) Fracture extending to apical third of root (distal) of 25.

The fracture in this case was considered to be a cracked tooth as it originated on the occlusal surface and extended onto the proximal surface and then onto the root surface until the apical third on both mesial and distal surfaces (Figures 2(a) and 2(b)).

## 3. Case Report 2

A female patient aged 51 years reported with severe, throbbing pain in left upper first premolar, that is, 24, since three days. The pain was aggravated on chewing and lingered on for few hours after removal of stimulus. There was history of sensitivity to cold beverages. The patient could recall episodes of similar pain with the same tooth for the past two years which was only temporarily relieved with desensitising tooth paste.

**Figure 3:**
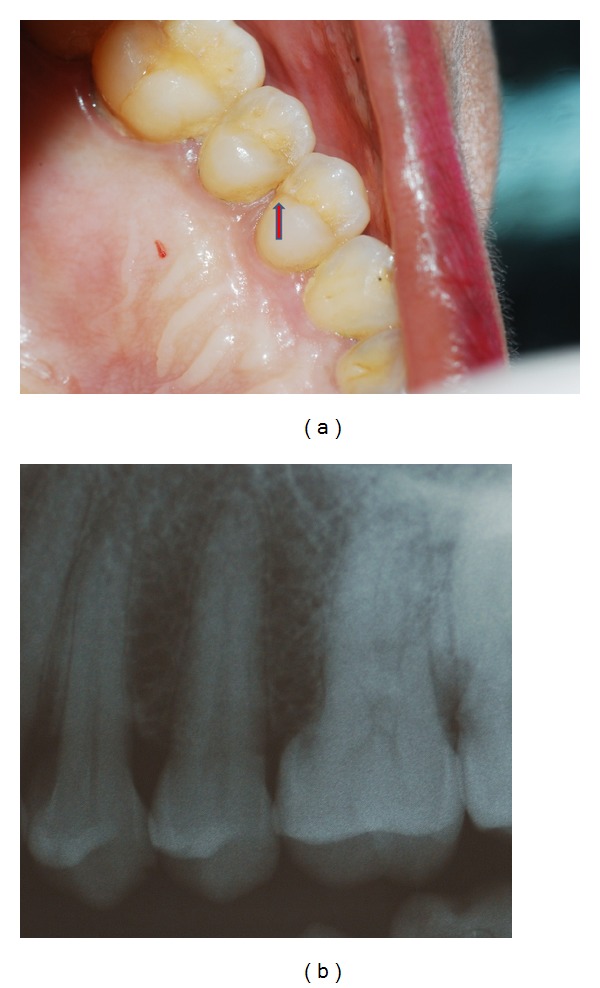
(a) Faint fracture line can be detected on distal marginal ridge extending onto proximal surface of 24. (b) Intraoral periapical radiograph of 24 reveals no significant findings.

**Figure 4:**
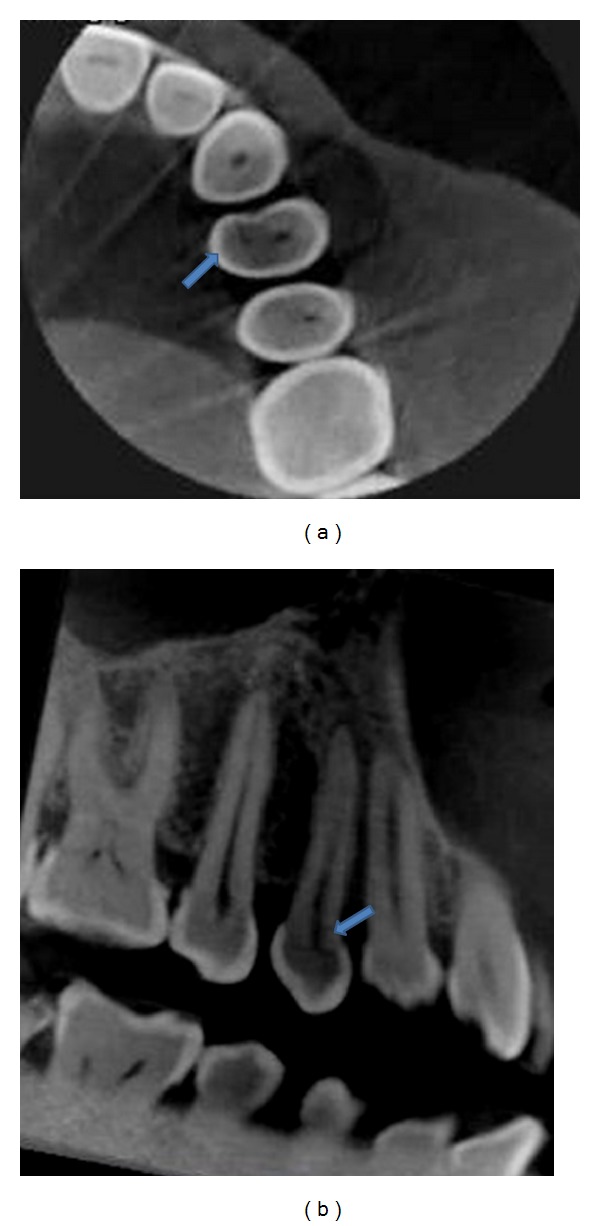
(a) Axial view of CBCT reveals a fracture line extending mesiodistally involving lingual pulp horn. (b) Sagittal view of CBCT reveals a fracture line extending mesiodistally at the level of pulp horn.

On clinical examination, no caries was detected nor restoration was present but a very faint craze line could be seen on the distal marginal ridge extending onto the distal proximal surface of 24 (Figure 3(a)). The extent of the craze line could not be determined. The tooth was tender on percussion, it did not exhibit mobility, and the fracture could not be separated using wedging forces. Periodontal probing depths were normal. Electric pulp test revealed immediate response. Radiograph did not confirm the fracture line or reveal any significant findings (Figure 3(b)). The patient was given a choice of using cone beam computed tomography (CBCT) to determine the extent of fracture and the prognosis of the tooth. She was explained the benefits and also assured that radiation exposure would be kept as minimal as possible. An informed consent was obtained from the patient. Cone beam computed tomography (Vatech, PaX-Reve 3D plus, pulse type generator, 5 × 5 cm field of view (FOV) and 0.08 mm voxel size) was used to determine the relative depth of fracture apically and the proximity of fracture to the pulp (Figures 4(a) and 4(b)). The fracture line was found to extend mesiodistally involving lingual pulp horn and apically it did not extend below the level of alveolar bone. Prognosis was considered to be favourable. Root canal treatment followed by crown restoration was then decided as most appropriate treatment plan. The suspicion of fracture extending to deeper aspects of root, coupled with acute symptoms of pain in the patient prompted the usage of CBCT for a clearer diagnosis, prognosis, and treatment plan.

## 4. Discussion

Cracked tooth is a distinct type of longitudinal fracture of the tooth and studies have indicated increased incidence of cracked tooth [8, 9]. This type of fracture is not only associated with complex and long standing restorations but also with minimally restored teeth and teeth without any restorations as noticed in case report 2. The teeth usually involved are mandibular molars (restored and nonrestored) followed by maxillary premolars and then by maxillary first molars [1, 10, 11]. Cracks in teeth are almost invariably mesiodistal fractures [12] although mandibular molars may occasionally fracture toward the facial or lingual surface. Longitudinal fractures are common in root canal-treated teeth, because the strength of root canal treated tooth has already been compromised by caries, restorations, or overextended access preparations [13] making it vulnerable to fracture. Crown restorations given in posterior teeth after endodontic therapy provide bracing effect and prevent crack initiation and propagation. In case report 1, failure to deliver crown restoration after root canal therapy may have led to the propagation of crack over a period of time leading to devastating results and finally extraction of tooth.

Etiological factors of these fractures are repeated biting on hard substances [2], prominent masticatory muscles [14], parafunctional habits, deep class I and II restorations, thermal stresses created by difference of coefficient of thermal expansion of restoration and dentin [15, 16], retentive pin placement with twist drills [17], and endodontically treated teeth.

Cracked tooth is a fracture which usually begins on the occlusal surface and grows from this surface toward the cervical surface and down the root. In case report 1 the fracture was present on centre of occlusal surface and progressed until the junction of middle third and apical third of the root. The more centred the fracture on the occlusal surface, the more it has a tendency to extend deeper before it shears toward the root surface. The deeper the fracture extends on to the root surface, the poorer the prognosis [7]. This fracture is considered “greenstick” because it is incomplete (either to the mesial or distal surface) or does not extend to the facial or lingual root surface [8]. Wedging forces produce no separable segments. In contrast, a split tooth has a fracture which is complete and extends to a surface in all areas [14]. A split tooth is usually the end result of a cracked tooth.

Cracked tooth is not a diagnosis by itself, but a finding. The objective is to first detect and then determine the extent of the fracture. Useful aids in detection of cracked tooth are transillumination [8], careful visualisation after removal of restoration, selective biting on objects such as the Tooth Slooth or Fracfinder, dental operating microscopes, staining, and wedging forces [3].

The relationship between fractures in the teeth and endodontic diagnoses depends upon the extent of the fracture. A cracked tooth may present with a variety of symptoms ranging from pain on biting, sensitivity to thermal changes, and mild to very severe, spontaneous pain consistent with irreversible pulpitis, pulp necrosis, or apical periodontitis [18]. Even an acute apical abscess, with or without swelling or a draining sinus tract, may be present if the pulp has undergone necrosis. Once the fracture has extended to and exposed the pulp, severe pulp and/or periapical pathosis may be present. This explains the variation in signs and symptoms and therefore the term “Cracked Tooth Syndrome” should not be used [7].

In a cracked tooth pulp and periapical tests also produce variable results. The pulp is usually responsive (vital) [14] but may be non-responsive (necrosis) as well. Directional percussion is also advocated. Percussion that separates the crack may cause pain due to stimulation of the periodontal ligament proprioceptors [7]. Periodontal probing is important and may disclose the approximate depth and severity of the fracture. However, subgingival fractures often do not create a probing defect. Therefore the absence of deep probing does not preclude a cracked tooth [19].

As the fracture in a cracked tooth is usually present in mesiodistal direction, it is not visible radiographically. Conventional dental radiography serves as an aid in assessing pulpal and periodontal compromise but gives little or no information on the direction and extent of the fracture. Depending on the extension towards the root and the relationship with the periodontium (below alveolar crest) the treatment is going to vary. If the fracture is limited to the crown surface, it can be restored. If a fracture extends below the alveolar crest, the prognosis is poor. Making the proper treatment decision is a challenge for the endodontist as there are limited noninvasive tools to assess the length of the fractures below the soft tissues and alveolar crest.

Newer methods of analysis, such as cone beam computed tomography (CBCT), are currently being studied in order to help identify longitudinal fractures in a nondestructive fashion [20]. The joint position statement by the American Association of Endodontists (AAE) and American Academy of Oral and Maxillofacial Radiology (AAOMR) regarding the use of CBCT in Endodontics states that “the patient's history and clinical examination must justify the use of CBCT by demonstrating that the benefits to the patient outweigh the potential risks [21]. Clinicians should use CBCT only when the need for imaging cannot be answered adequately by lower dose conventional dental radiography or alternate imaging modalities.” Every effort should be made to reduce the effective radiation dose to the patient for endodontic-specific tasks. Using the smallest possible field of vision (FOV), the smallest voxel size, the lowest mA setting, and the shortest exposure time in conjunction with a pulsed exposure mode of acquisition is recommended [21].

In case report 2 we have used multi-slice view (thickness 1 mm, spacing 0.4 mm) of CBCT to detect the fracture at various planes and to determine the apical extent as well as proximity to the pulp and predict the prognosis. The volume of data acquired in a single scan allows visualizing a tooth from any coronal, sagittal, or axial view, and the ability to reslice the volume at any slice thickness. This volume of data can be manipulated repeatedly to gather large amount of information about the tooth and its periapical tissues.

Mora et al. [20] and Valizadeh et al. [22] in their in vitro study found local CT and CBCT, respectively, to be more efficient in detection of longitudinal tooth fractures compared to conventional dental radiographs.

Treatment of a cracked tooth depends on the nature (depth and location) of the fracture. If there are no symptoms of irreversible pulpitis, a crown may be placed. Some of these teeth may eventually manifest irreversible pulpitis or pulp necrosis [23]. In that case root canal treatment can be performed through the crown. After endodontic access, the pulp chamber floor is examined carefully; transillumination is a useful aid during this procedure. If the fracture extends through the chamber floor, further treatment is usually hopeless and extraction is preferred [24]. An exception is the maxillary molar, which may be hemisected along the fracture, saving half (or both halves) of the crown and supporting roots. If a partial fracture of the chamber floor is detected, the crown may be bound with an orthodontic band or temporary crown to protect the cusps until final restoration is performed [25]. This also helps to determine whether symptoms decrease during root canal treatment [26]. The rationale employed for treatment of cracked tooth is that if pain symptoms are not relieved, the prognosis is significantly poorer, and extraction may be necessary. The overall prognosis depends on the situation but is always questionable at best. The patient is informed about the possible outcomes and the unpredictability of the duration of treatment. The fracture may continue to grow and become a split tooth, with devastating consequences requiring tooth extraction or additional treatment. Tan et al. [19] studied a small number (*n* = 50) of root-filled cracked teeth with a diagnosis of irreversible pulpitis and determined a 2-year survival rate of 85.5%. Krell and Rivera [23] evaluated 127 patients with teeth diagnosed with reversible pulpitis that had a cracked tooth in which the treatment was placement of a crown restoration without performing root canal treatment. Twenty percent of these cases turned into irreversible pulpitis or necrosis within 6 months and required root canal treatment, but none of the other teeth required root canal treatment over the 6-year evaluation period. In case report 1 the failure to detect the crack and determine its severity, followed by failure to place a crown restoration after root canal treatment, may have led to crack propagation and poor prognosis of the tooth. In case report 2 early detection of extent and severity of the crack with the aid of CBCT helped in improving the overall prognosis of the tooth.

## 5. Conclusion

Early detection, diagnosis, and prompt treatment are the key to the successful management of a cracked tooth. Careful attention to signs and symptoms and using conventional techniques and advanced methods like CBCT along with timely treatment can limit the crack propagation. Assessment of the pattern or extent of fracture with the aid of CBCT can help the endodontist in making a decision about root canal treatment or extraction and reassuring the patient why root canal treatment or extraction is necessary for such a faint fracture line. It also helps in having reliable documentation and strong evidence for future reference. Nevertheless, the higher dose of radiation should be kept in mind and hence should be reserved for only challenging cases after thorough examination.

## References

[1] Walton RE, Walton RE (1995). Longitudinal tooth fractures. *Torabinejad M. Principles and Practice of Endodontics*.

[2] Rivera EM, Williamson A (2003). Diagnosis and treatment planning: cracked tooth. *Texas dental journal*.

[3] Rivera EM, Walton RE Cracking the cracked tooth code: detection and treatment of various longitudinal tooth fractures.

[4] Bader JD, Martin JA, Shugars DA (1995). Preliminary estimates of the incidence and consequences of tooth fracture. *Journal of the American Dental Association*.

[5] Schetritt A, Steffensen B (1995). Diagnosis and management of vertical root fractures. *Journal of Canadian Dental Association*.

[6] Tamse A, Zilburg I, Halpern J (1998). Vertical root fractures in adjacent maxillary premolars: an endodontic-prosthetic perplexity. *International Endodontic Journal*.

[7] Rivera EM, Walton RE (2007). Longitudinal tooth fractures: findings that contribute to complex endodontic diagnoses. *Endodontic Topics*.

[8] Abou-Rass M (1983). Crack lines: the precursors of tooth fractures—their diagnosis and treatment. *Quintessence International*.

[9] Ehrmann EH, Tyas MJ (1990). Cracked tooth syndrome: diagnosis, treatment and correlation between symptoms and post-extraction findings. *Australian Dental Journal*.

[10] Nair MK, Nair UDP, Gröndahl HG, Webber RL, Wallace JA (2001). Detection of artificially induced vertical radicular fractures using Tuned Aperture Computed Tomography. *European Journal of Oral Sciences*.

[11] Van Daatselaar AN, Dunn SM, Spoelder HJW (2003). Feasibility of local CT of dental tissues. *Dentomaxillofacial Radiology*.

[12] Weine FS, Dewberry JA, Weine FS (1982). Cracked-tooth syndrome: vertical fractures of posterior teeth. *Endodontic Therapy*.

[13] Eakle WS, Maxwell EH, Braly BV (1986). Fractures of posterior teeth in adults. *The Journal of the American Dental Association*.

[14] Cameron CE (1976). The cracked tooth syndrome: additional findings. *The Journal of the American Dental Association*.

[15] Brown WS, Jacobs HR, Thompson RE (1972). Thermal fatigue in teeth. *Journal of Dental Research*.

[16] Eakle WS (1986). Effect of thermal cycling on fracture strength and microleakage in teeth restored with a bonded composite resin. *Dental Materials*.

[17] Standlee JP, Collard EW, Caputo AA (1970). Dentinal defects caused by some twist drills and retentive pins. *The Journal of Prosthetic Dentistry*.

[18] Brynjulfsen A, Fristad I, Grevstad T, Hals-Kvinnsland I (2002). Incompletely fractured teeth associated with diffuse longstanding orofacial pain: diagnosis and treatment outcome. *International Endodontic Journal*.

[19] Tan L, Chen NN, Poon CY, Wong HB (2006). Survival of root filled cracked teeth in a tertiary institution. *International Endodontic Journal*.

[20] Mora MA, Mol A, Tyndall DA, Rivera EM (2007). In vitro assessment of local computed tomography for the detection of longitudinal tooth fractures. *Oral Surgery, Oral Medicine, Oral Pathology, Oral Radiology and Endodontology*.

[21] (2011). AAE and AAOMR joint position statement. Use of cone-beam-computed tomography in endodontics. *Pennsylvania Dental Journal*.

[22] Valizadeh S, Khosravi M, Azizi Z (2011). Diagnostic accuracy of conventional, digital andCone Beam CT in vertical root fracture detection. *Iranian Endodontic Journal*.

[23] Krell KV, Rivera EM (2007). A six year evaluation of cracked teeth diagnosed with reversible pulpitis: treatment and prognosis. *Journal of Endodontics*.

[24] Türp JC, Gobetti JP (1996). The cracked tooth syndrome: an elusive diagnosis. *Journal of the American Dental Association*.

[25] Pane ES, Palamara JEA, Messer HH (2002). Stainless steel bands in endodontics: effects on cuspal flexure and fracture resistance. *International Endodontic Journal*.

[26] Zimet PO, Endo C (2000). Preservation of the roots—management and prevention protocols for cracked tooth syndrome. *Annals of the Royal Australasian College of Dental Surgeons*.

